# Brooding in the Chilean Oyster *Ostrea chilensis*: Unexpected Complexity in the Movements of Brooded Offspring within the Mantle Cavity

**DOI:** 10.1371/journal.pone.0122859

**Published:** 2015-04-13

**Authors:** Daniela A. Mardones-Toledo, Jaime A. Montory, Alyssa Joyce, Raymond J. Thompson, Casey M. Diederich, Jan A. Pechenik, Maria L. Mardones, Oscar R. Chaparro

**Affiliations:** 1 Instituto de Ciencias Marinas y Limnológicas, Universidad Austral de Chile, Valdivia, Chile; 2 Department of Biological & Environmental Science, University of Gothenburg, Gothenburg, Sweden; 3 Ocean Sciences Centre, Memorial University of Newfoundland, NFLD, Saint John´s, Canada; 4 Biology Department, Tufts University, Medford, 02155, Massachusetts, United States of America; GEOMAR Helmholtz Centre for Ocean Research Kiel, GERMANY

## Abstract

Brooding in invertebrates serves to protect embryos from stressful external conditions by retaining progeny inside the female body, effectively reducing the risk of pelagic stages being exposed to predation or other environmental stressors, but with accompanying changes in pallial fluid characteristics, including reduced oxygen availability. Brooded embryos are usually immobile and often encapsulated, but in some *Ostrea* species the embryos move freely inside the female pallial cavity in close association with the mother’s gills for as long as eight weeks. We used endoscopic techniques to characterize the circulation pattern of embryos brooded by females of the oyster, *Ostrea chilensis*. Progeny at embryonic and veliger stages typically circulated in established patterns that included the use of dorsal and ventral food grooves (DFG, VFG) to move anteriorly on the gills. Both embryos and veligers accumulated around the mother’s palps, and remained there until an active maternal countercurrent moved them to the gill inhalant area. Both food grooves were able to move embryos, veligers, and food-particle aggregates anteriorly, but the DFG was more important in progeny transport; early embryos were moved more rapidly than veligers in the DFG. A microcirculation pattern of embryos was apparent when they were moved by gill lamellae: when they were close to the VFG, most embryos lost gill contact and ´´fell´´ down to the DFG. Those that actually reached the DFG moved anteriorly, but others came into contact with the base of the lamellae and again moved towards the VFG. The circulation pattern of the progeny appears well-suited for both cleaning them and directing them posteriorly to an area where there is more oxygen and food than in the palp region. This process for actively circulating progeny involves the feeding structures (gill and palps) and appears to be energetically costly for the female. It also interferes with feeding, which could explain the poor energy balance previously documented for brooding females of this species.

## Introduction

The physical protection of embryos by maternal brooding is well documented in several species of aquatic invertebrates [1, 2, 3, 4, 5, 6]. As a developmental strategy, brooding may reduce predation or protect larvae from unfavorable environmental conditions at crucial periods of early development [7, 8, 9, 10].

Brooding in invertebrates frequently reduces embryo mobility by restricting them to particular areas of the maternal cavities [11]. Embryos may be retained inside of marsupial gills [12], anchored with stalks [13], or enclosed within capsules [3, 14]. However, in some species the embryos remain mobile within the maternal mantle cavity [15, 16]. Oyster embryos in the genus *Ostrea*, for instance, are free to move throughout the infrabranchial (inhalant) chamber of the mantle cavity [17, 18, 19, 20]. However, the extent of embryo incubation and the duration of pelagic life after veligers are released from the mother differs considerably among species of this genus. For example, the puelchana oyster (*Ostrea puelchana*) releases veligers in an early stage of development, after only 3–9 days of incubation [21, 22]. In contrast, many commonly farmed flat oysters, including the European oyster (*O*. *edulis*), the Olympia oyster (*O*. *lurida*), and the Australian native flat oyster (*O*. *angasi*) brood their embryos for shorter periods (e.g. one to two weeks depending on water temperature) and release veliger larvae that then also have a relatively long pelagic phase of up to three weeks [23, 24, 25].

Chilean populations of the flat oyster (*Ostrea chilensis*) are exceptional in that they brood their young for almost the entire developmental period. Though brooding periods in some *O*. *chilensis* populations kept under hatchery conditions in New Zealand have been as short as three weeks [26], embryos of *O*. *chilensis* are generally brooded for approximately eight weeks [27, 28]. Consequently, the females of this species release unusually large and well-developed pediveliger larvae with an extremely short pelagic phase of only a few minutes to 48 h [4]. Additionally, *O*. *chilensis* females brood a high number of offspring (embryos per female: 3,200–113,000, mean value of 58,000 for early stages, [27, 29]), and all brooded offspring are at the same developmental stage within a given female.

The embryos’ relationship with the maternal gills and labial palps is of particular interest in brooding *O*. *chilensis*, because the presence of larvae could interfere with filter feeding, including particle capture, transport, and selection [16]. Indeed, females have been shown to have reduced clearance rates when brooding [30], which limits their energy availability. Energetic growth models for *O*. *chilensis* have shown that females at the end of the brooding period have significantly lower tissue mass than non-brooding females, a fact that demonstrates the considerable energetic costs to the mother associated with such a long brooding period [30].

Though brooding has been shown to be energetically costly to female *O*. *chilensis*, the processes that take place in the female pallial cavity during brooding are largely unknown, particularly those that relate to food particle transport and the female management of early embryonic stages. In *O*. *chilensis*, the circulation pattern of advanced veliger larvae in the mantle cavity has only been partially characterized. It appears that larvae travel inside the brooding cavity using the same food grooves that transport food to the female’s mouth [16]. Embryos move towards the anterior end of the female pallial cavity, reaching the palps, where particle selection occurs before ingestion of food [31, 32, 33, 34]. Accordingly, the physical presence of numerous veligers between the demibranchs and palps may directly alter maternal feeding processes. Advanced veligers of *O*. *chilensis* can also consume the food particles, which may further reduce food availability for females [16, 28].

In *O*. *chilensis*, prior endoscopic observations noted that a cyclical movement of advanced-stage veligers concentrates them around the female palps before returning them to the posterior inhalant region, where they recommence their journey to the palps [16]. The physical process of re-dispersion of accumulated veligers around the palps is not clearly understood. However, such transport is presumably associated with a maternally generated current that redistributes veligers to a region of increased oxygen and food supply in proximity to the inhalant area [16]. Whether a similar pattern occurs with non-swimming, early embryos is unknown.

The maternal role in transporting food, embryos, and veligers within the mantle cavity of *O*. *chilensis* females has not been sufficiently investigated. Considering the long brooding period in *O*. *chilensis*, it is particularly important to determine the role that the mother plays in progeny movement at different larval developmental stages and the impact of such movement on food transport and particle selection prior to ingestion. In this study, we investigated these processes by using endoscopic observations in brooding and non-brooding oysters in order to determine the relationships between females and embryos during the entire brooding period. We hypothesized that brooding females would use the gill and food grooves differently for transporting food particles and embryos, and that their use would depend on both the female reproductive condition (brooding or not brooding) and the developmental stage of the embryos.

## Materials and Methods

### Sampling and maintenance of oysters

Adult oysters (4–5 cm shell length) were collected from the Rio Quempillén estuary of Chiloé, in Southern Chile (41°52'S, 73°46'W) and transported immediately to the laboratory. We collected individuals during two key periods: March-April 2013 when *O*. *chilensis* was unlikely to be brooding, and November-December 2013 during the reproductive period [4]. In the lab, oysters were fed with the cultured microalgae *Isochrysis galbana* and maintained for several days in aquaria with daily seawater changes (salinity: 30–32 psu, temperature: 17–18°C).

### Endoscopy of the brood cavity

A perforation of 3–4 mm diameter was cut in the right margin of each oyster’s shell [16]. In some females, an anterior hole through the shell was made near the labial palps, while in others a second hole was made in the inhalant area to provide direct access of the endoscopy tip to both palps and to female gills. For brooding females, the holes were temporarily sealed with parafilm before endoscopic analysis to prevent loss of embryos or veligers.

Before making our observations, individual oysters were placed in small aquaria (1 L) filled with seawater and a phytoplankton suspension of 30,000 cells ml^-1^ of *I*. *galbana*, at the same conditions of salinity and temperature already described. For observations of the pallial cavity, the 2.5 mm diameter tip of an endoscope (Olympus OTV SX model) was inserted through the appropriate hole in the shell. Movement of the endoscope inside the pallial cavity was facilitated by using a micromanipulation system. A xenon light source provided cold light to the endoscope; the resulting images were stored on a digital DVR recorder. The endoscope tip remained inserted in the pallial cavity of each female oyster for at least 15 min, until pumping activity was identified. Endoscopic observations were facilitated by adding an extra aliquot of microalgae near the hole made in the oyster shell. Filming commenced once pumping was initiated, and usually continued for more than 0.5 h for each oyster.

**Fig 1:**
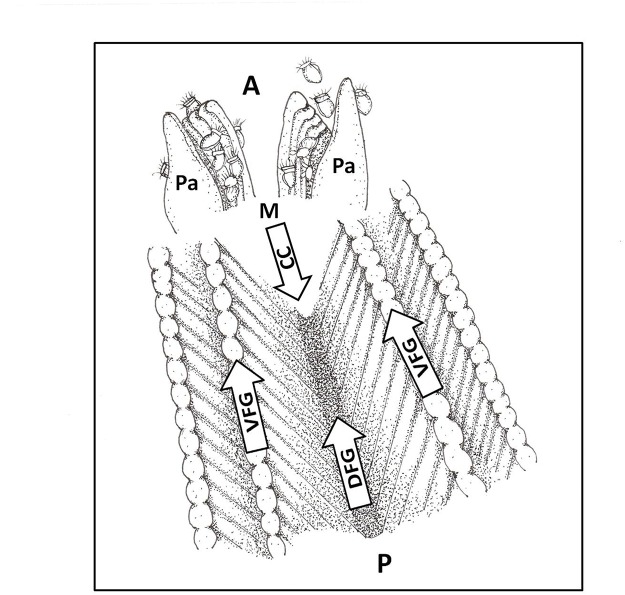
Ostrea chilensis. Diagram of the pallial cavity of a brooding female. Note the veligers that have accumulated near the female palps. A: anterior, CC: countercurrent, DFG: dorsal food groove, M: mouth, P: posterior, Pa: female palps, VFG: ventral food groove.

Endoscopic observations in the female pallial cavity were focused on the transport of embryos and algal aggregations along the dorsal and ventral food grooves (Fig 1), the movement of embryos along the face of the gill lamellae toward the ventral food groove, actions of the labial palps on the progeny cleaning process, and maternal counter currents in the repositioning of embryos and veligers.

### Transport of particle aggregations and progeny through the food grooves

#### Velocity and size measurements

A suspension of *Isochrysis galbana* was added to the tank at a concentration of approx. 30,000 cells ml^-1^. During the endoscopy filming process, small pulses of a solution of nontoxic, light-reflective red plastic particles (2–10 μm diameter) were added to facilitate identification of microalgal movements in the endoscopy images [16].

The velocities of food and progeny moving through the dorsal (DFG) and ventral (VFG) food grooves were estimated through sequential image processing by measuring distance traveled against time (number of filming 'frames'). The developmental stages studied were embryos (without shells or very early shelled, after 2–4 weeks of incubation, mean size ± SD of 275 ±28 μm, N = 80), or veligers (after 6–7 weeks of incubation, mean shell size ± SD of 432 ± 31 μm, N = 81). The total brooding period has been recorded to be about 8 weeks [4]). The distance traveled by the algal aggregations or progeny was estimated using reference size measurements made on pieces of gills taken from the experimental oysters [35, 36]. Gill measurements, made using an inverted Zeiss microscope, were taken of the plicae size at the gill base near the DFG, in mid-sections of the filaments, and at the distal end near the ventral groove and measured in a frontal view of the lamellae. Also, we measured the gill plicae size from a frontal view of the VFG.

The use of gill references for size depended on the location and/or direction of approach of the endoscope from the object (microalgae or progeny). For brooding females, the base or top of the gill filament was measured and used as a size reference when determining velocities in the DFG and VFG, respectively), as well as the mean size of brooded progeny. For this, after endoscopic filming, a sample of oyster progeny was obtained and the size measurements were made using an inverted Zeiss microscope.

#### Female countercurrent

Velocity was estimated for both embryos and veligers concentrated in the maternal palp region as they were being directed posteriorly toward the maternal mantle cavity. This movement is hereafter identified as the 'female countercurrent'. The velocity was measured as previously described: sequential frames from the recorded video were analyzed and the distance travelled by embryos was estimated using the gill plicae size as a reference. Average speeds were based on 4 to 6 measurements from each of 10 females brooding embryos and 11 females brooding veligers. Since the counter-current flow is not constant, we measured the counter-current velocity several times for each individual female.

In some cases, embryos moved towards the VFG of the gill across the lamellae and fell back towards the dorsal groove before reaching the ventral food groove. This movement is hereafter referred to as "falling" and was measured using techniques described above.

#### Use of food grooves

The transport of food aggregations in food grooves was examined for oysters collected during brooding and non-brooding periods in order to examine differential use of the food grooves for particle transport.

The importance of these grooves (DFG and VFG) in transporting food and/or progeny was estimated by recording the number of embryos and larvae, or aggregates of microalgae, moved per unit time. It was also possible to observe and identify aggregations (microalgae plus gill mucus) as a unit, given the magnification of the endoscope.

### Relationships between female labial palps and embryos

Endoscopic observations were made directly in the region of the female labial palps for both embryo and veliger stages. In these observations, we studied the mother-progeny dynamics and maternal behavior, especially in relation to the cleaning process involving the palps.

### Statistical analysis

After data normality and homogeneity of variance were confirmed, we used one-way ANOVA to identify differences in particle transportation and variations in particle transport velocity between the DFG and VFG for both females during the non-brooding period and for brooding and non-brooding females during the brooding period. In addition, one-way ANOVA was used to identify differences in transport velocity of embryos compared to veligers as they moved through the female lamella to the VFG. In the same way, we compared velocity of embryos when moving to the VFG with their ″falling″ velocity to the DFG after they lost physical contact with the gill filaments, and we compared the female countercurrent velocity between early and advanced embryos.

We used two-way ANOVA to test the utilization of DFG and VFG in transporting both food and progeny by females brooding either embryos or veligers. During the brooding period, the same test was used to determine differences in transport velocity of particles, embryos, and veligers, again comparing the DFG with the VFG. When significant differences between treatments were identified, *a posteriori* Tukey tests were used to determine where the differences lay. In all analyses, we used a significance level of 0.05 to determine whether differences were statistically significant [37].

### Ethics Statement

The model species used in the present study is not an endangered species (IUCN Red Data Books), and according the National Fishery Service is not on the protected species list. During most of the year, the flat oyster is harvested from natural banks without restrictions another than size and reproductive condition. According to local current regulations, those rules do not apply to oyster culture stations, which is where the individuals used in our experiments were obtained.

## Results

### Transport of particle aggregations and progeny in food grooves

#### Non-brooding period

**Fig 2:**
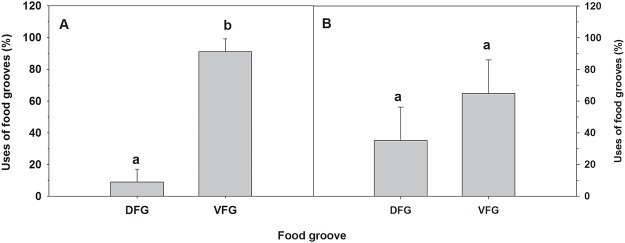
Ostrea chilensis. Food transport within the gill food grooves by adult oysters (A) non-brooding period and (B) brooding period. DFG: Dorsal food groove; VFG: Ventral food groove. Letters above the bars indicate significant differences (p<0.05); mean + SD shown.

Adult females transported over 85 ± 8.1% of food aggregates anteriorly through the ventral food groove (VFG) (Fig 2A), indicating that during non-brooding periods, this is the main food transport system from the gill to the mouth (One-way ANOVA, F _(1,30)_ = 340.1; p = 0.00001). The particles carried in the VFG were first collected on the frontal lamellar surface and then moved towards that groove. The particles that reached the dorsal food groove (DFG) entered there via the inhalant flow rather than being transported through the lamellae.

#### Brooding period

Non-brooding females transported approximately 64 ± 21.2% of food aggregations along the VFG (Fig 2B). Though non-brooding females used the VFG to transport food aggregations more than they used the DFG, the differences in use of the grooves was not statistically significant (One-way ANOVA F _(1,15)_ = 4.95; p = 0.056).

**Fig 3:**
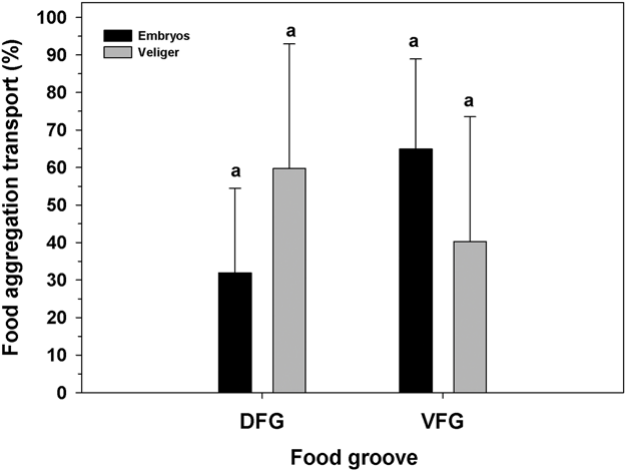
Ostrea chilensis. Utilization of ciliated grooves to move food particles by oyster females brooding progeny at different developmental stages (early: embryos or advanced: veliger stages). DFG: Dorsal food groove; VFG: Ventral food groove. Different letters above the bars indicate significant differences (p<0.05); mean + SD shown.

**Fig 4:**
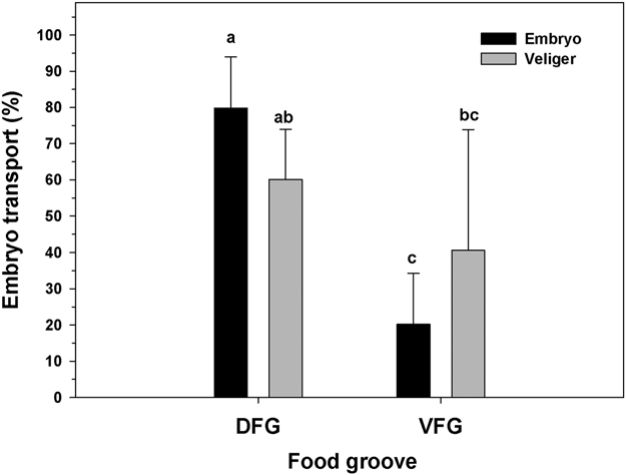
Ostrea chilensis. Utilization of female oyster ciliated grooves to transport embryo or veligers. DFG: Dorsal food groove; VFG: Ventral food groove. Different letters above the bars indicate significant differences (p<0.05); mean + SD shown.

Brooding females moved both food and embryos along both food grooves. In these females, the utilization of the DFG (46 ± 21.8% of total food aggregations) and VFG (53 ± 23.2% of total food aggregations) to move food aggregations was similar, independent of the state of development of the brooded progeny (Fig 3; Two-way ANOVA, F_(1,30)_ = 2.67; p = 0.133). However, the use of the DFG and VFG for transport of progeny showed significant differences depending on the developmental stage of the brooded offspring (Fig 4; Two-way ANOVA, F_(1,30)_ = 7.49; p = 0.0021). The DFG was utilized significantly more often (79.81 ± 14.14%) than the VFG (20.19 ± 16.21%) only when early embryos were present, while the use of these grooves was similar (DFG = 60.09 ± 13.86%., VFG = 40.65 ± 33.24%) when advanced veligers were present (Fig 4).

### Transport velocity for food and progeny

#### Transport of food

**Fig 5:**
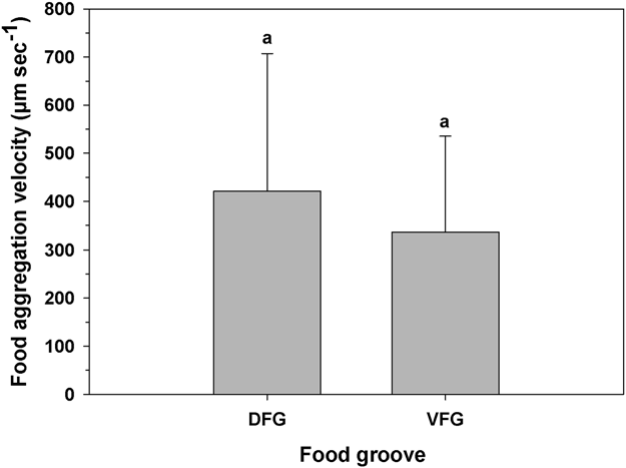
Ostrea chilensis. Velocity of food particle aggregations in different grooves transported to the mouth in oyster females during the non-brooding period. DFG: Dorsal food groove; VFG: Ventral food groove. Different letters above the bars indicate significant differences (p<0.05); mean + SD shown.

During the non-brooding period, there were no significant differences in mean particle velocities along the two food grooves (One-way ANOVA, F _(1,115)_ = 2.701; p = 0.103) with average values (mean ± SD) of 421.10 ± 285.90 and 336.33 ± 199.35 μm sec^-1^ in the DFG and VFG, respectively (Fig 5).

**Fig 6:**
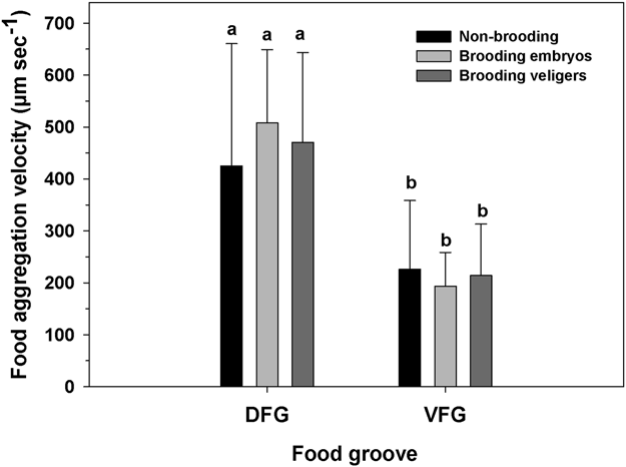
Ostrea chilensis. Velocity of food particles in the food grooves of oyster females that were not-brooding, brooding embryos, or brooding veligers during the reproductive period. DFG: Dorsal food groove; VFG: Ventral food groove. Different letters above the bars indicate significant differences (p<0.05); mean + SD shown.

However, during the brooding period, food aggregations moved more than twice as fast (Two-way ANOVA, F_(1,133)_ = 76.443; p = 0.001, Fig 6) in the DFG (450 ± 91 μm sec^-1^) than in the VFG (205 ± 72 μm sec^-1^) (Fig 6). Within each groove, though, mean food particle velocity did not differ significantly among early embryo-brooding females, veliger-brooding females, and non-brooding females (Fig 6; Two-way ANOVA, F_(2,133)_ = 1.274; p = 0.283).

#### Transport of brooded offspring

**Fig 7:**
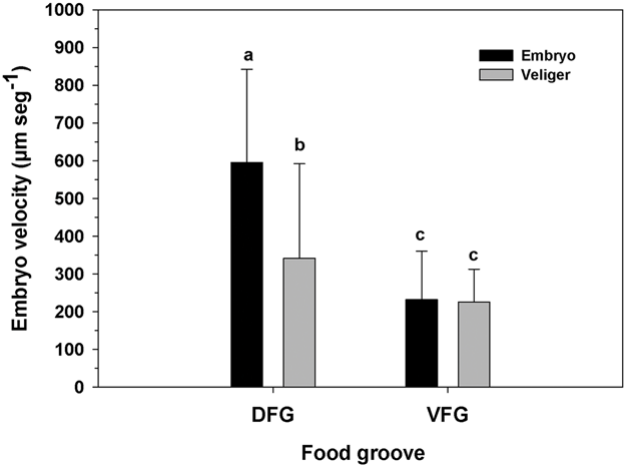
Ostrea chilensis. Velocity of embryos and veligers through the dorsal and ventral food grooves in brooding females. DFG: Dorsal food groove; VFG: Ventral food groove. Different letters above the bars indicate significant differences (p<0.05); mean + SD shown.

The velocity of offspring along the food grooves of brooding *O*. *chilensis* females depended on the groove that offspring were in (Fig 7; Two-way ANOVA, F _(1,161)_ = 57.548; p = 0.001), and the stage of embryonic development (Fig 7; Two-way ANOVA, F_(1,161)_ = 20.088; p = 0.001); the interaction between the two variables was also significant (Two-way ANOVA, F_(1,161)_ = 12.111; p = 0.002). Progeny always moved faster in the DFG than in the VFG, but in the DFG, embryos (595.34 ± 247.04 μm sec^-1^) moved far more quickly than veligers did (341.68 ± 250.24 μm sec^-1^), while in the VFG the speed of embryos (232.04 ± 128.17 μm sec^-1^) and veligers (225.96 ± 86.48 μm sec^-1^) was similar.

**Fig 8:**
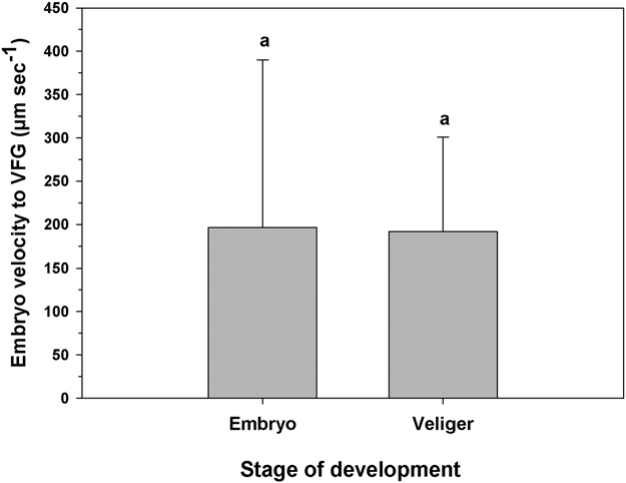
Ostrea chilensis. Velocity at which offspring were moved along the female lamella towards the ventral food groove. VFG: Ventral food groove. Different letters above the bars indicate significant differences (p<0.05); mean + SD shown.

The veligers moved on the face of the lamellae toward the VFG with an average velocity of 191.91 (± 109.08) μm sec^-1^, which was not significantly different from the velocity of embryos making the same movements (Fig 8; One-way ANOVA, F_(1,30)_ = 0.005; p = 0.94). The movement of progeny using the lamellae to reach the DFG occurred much less frequently than to reach the VFG.

**Fig 9:**
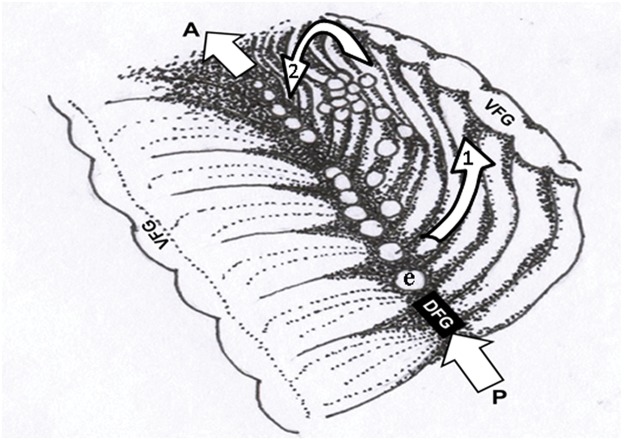
Ostrea chilensis. Schematic of the embryo microcirculation pattern inside the female pallial cavity. 1: embryo movement on the female lamella to VFG, 2: loss of embryo contact with the female gill lamella and their subsequent fall to the DFG. e: embryos, P: posterior female region, A: anterior female region, VFG: ventral food groove, DFG: Dorsal food groove.

In females that were brooding embryos, the demibranch usually had a concave inner surface and a corresponding convex outer surface (Fig 9).

**Fig 10:**
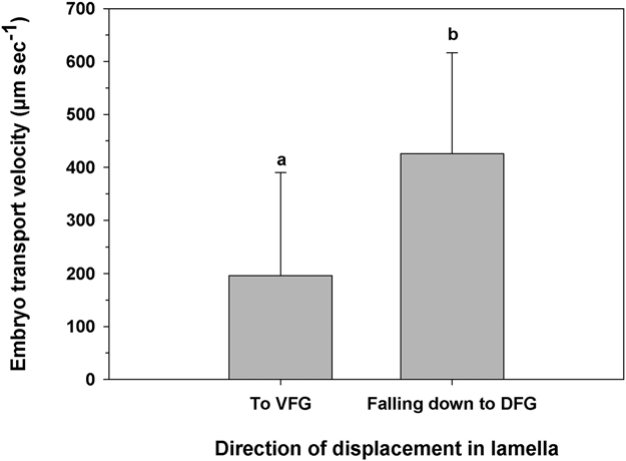
Ostrea chilensis. Velocity of embryos on frontal lamellae moving toward the VFG and the velocity at which embryos fall to the DFG after they lose contact with the gill lamella. VFG: ventral food groove; DFG: Dorsal food groove. Different letters above the bars indicate significant differences (p<0.05); mean + SD shown.

Embryos moved on the lamella along the convex surface with an average velocity of 196.55 ± 193.57 μm sec^-1^ (mean ± SD) (Fig 10). Movements of embryos on the concave surface were also seen, but less often. Most of the embryos that travelled on the convex lamella lost contact with the gill filaments and ″fell down″ toward the DFG. This descent occurred with an average velocity of 425.94 ± 190.47 μm sec^-1^, which was significantly higher (One-way ANOVA, F_(1,30)_ = 6.752; p = 0.018) than the transport velocity via the lamella to the VFG (Fig 10). Some of the embryos that descended and made contact with the DFG moved anteriorly. However, before most of the embryos could complete their descent to the DFG, they were driven by water flow to the middle region of the front surface of the lamella, where they again began their movement along the gill filaments toward the VFG. This mini-cycle lasted for varying amounts of time, unless the embryos 1) were retained in the DFG and moved anteriorly to the region of the palps; 2) reached the VFG itself and were then moved toward the palps or 3) fell from the VFG to the dorsal region across the demibranchs. The latter situation was quite common in early embryos and occurred most often in the distal half of the gill associated with the inhalant region. Moreover, when early embryos did reach the VFG in the anterior half of the gill, near the palps, they were moved using this groove until they reached the female palps.

#### Maternal countercurrents

**Fig 11:**
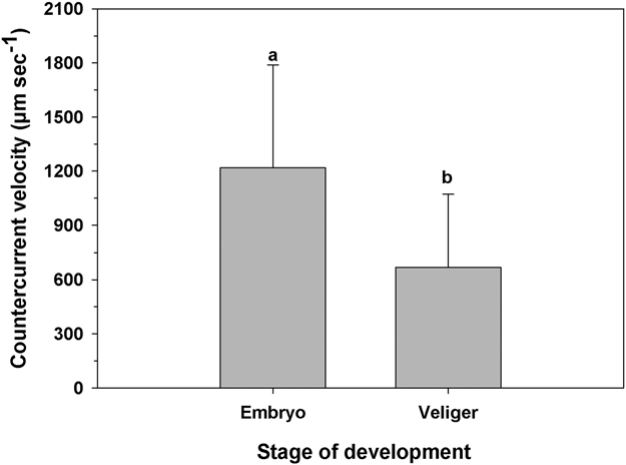
Ostrea chilensis. Velocity of female-generated countercurrents, which transport progeny from labial palps to the posterior mantle cavity for embryos and veligers. Different letters above the bars indicate significant differences (p<0.05); mean + SD shown.

Embryos that accumulated near the labial palps were sporadically dispersed and relocated to the posterior region of the maternal mantle cavity, at a velocity of 1,218.58 ± 570.47 μm sec^-1^, by the female countercurrent (Fig 11). For females incubating veligers (which were moved by the countercurrent much more often), the corresponding velocity was 668.52 ± 403.24 μm sec^-1^ (Fig 11), which was significantly slower than the velocity of embryos (One-way ANOVA, F_(1,30)_ = 6.61; p = 0.018).

### Relation between female labial palps and progeny

#### Embryos

A constant supply of embryos approached the female labial palp region via the VFG, typically with an attached mucus cord. This facilitated aggregation of embryos that passed from the VFG and reach the middle region or the distal tips of the maternal labial palps. These aggregations broke down between the adjacent palps in a maternal cleaning process so that embryos and mucous aggregations were released independently. Embryos were reintegrated into the female mantle cavity, and reached the anterior region near the female mouth while food items sorted from the mucus strings could enter the mouth. In contrast, no embryos that moved along the DFG were observed to reach the palps. When they reach the anterior part of the female, embryos move to the VFG using the gill filaments, and from this groove, embryos reach the female labial palps.

#### Veligers

These more advanced stages also approached the labial region via the VFG and could have some mucous attached to them, but, unlike embryos, they were not generally aggregated. As the veligers possess cilia, they could remain suspended between the labial palps while still moving and rotating for various periods of time before being dispersed and relocated by the maternal countercurrent. However, as with embryos, the veligers could still be subjected to a maternal ‘cleaning’ process. Small aggregations of advanced veligers that sometimes reached the palps were also separated from the attached mucus but typically this occurred in the distal region of the palps.

## Discussion

In brooding bivalve molluscs, the mantle cavity frequently retains embryos and larvae within an infrabranchial area that is not only important for capture of food particles [38, 39, 40, 41], but also for gas exchange [42]. Food particles caught in the gill are moved towards the oral region for ingestion or alternatively for disposal as pseudofeces. The dorsal and ventral food grooves are responsible for the transport of captured particles to the anterior region of the bivalve. However, the importance of each groove appears to be species-specific [35, 36] and the utilization of the grooves generally does not vary over time; the extent of their utilization depends upon the gill type (heterorhabdic or homorhabdic). *Ostrea chilensis* is novel in that females have differentiated the use of the food grooves depending on whether they are in the reproductive period or the non-reproductive period. During the non-breeding period, the VFG carries more than 85% of the food aggregations to the mouth. During the brooding period, however, the dorsal and ventral food grooves are equally important for transporting food, whether or not the females are brooding at that time (Fig 2). Increased participation of the DFG in food transport during the reproductive period could be related to possible development of cilia prior to brooding (a subject that warrants more thorough study) to promote the movement of embryos and food particles. The increased importance of the DFG could be caused by a relative reduction in the transport capacity of the VFG, which has an active role in transporting larger advanced veligers, a situation that could reduce transport capabilities of the food aggregates.

Prior calculations of particle transport speeds in others bivalve species provide an interesting comparison with the velocities recorded in this study. In some oyster species the DFG showed higher transport velocities than the VFG (e.g. *Crassotrea virginica*; 329 to 406 for VFG, 1244 to 1425 μm sec^-1^ for DFG; *Crassotrea gigas* 594 to 630 in the VFG, 1001–1110 μm sec^-1^ in the DFG)[36]. In the freshwater mussel *Dreissena polymorpha*, particles can be transported by both the marginal food groove of the inner demibranchs and the ciliated dorsal tracts, with average speeds of 156 μm sec^-1^ and 152 μm sec^-1^, respectively [40]. In other species, all of them with homorhabdic gills, particle transport is done only by the VFG (e.g. *Mytilus edulis*, [43]; *Mya arenaria* and *Mytilus edulis*, [44]; *Mytilus edulis* and *Mytilus trossulus*, [36]) and with varying speeds, but at higher speeds on the gills of epibenthic species (e.g. *Mytilus chilensis*, approximately 210–240 μm sec^-1^, *Venus antiqua*, approx. 40 μm sec ^-1^, *Mulinia edulis* and *Tagelus dombei*, approx. 150 μm sec^-1^, [41]). Compared with these other species, we found that *O*. *chilensis* transported food particles along the VFG at very high rates (non-brooding season, 336 μm sec^-1^), which was even slower than the rate at which particles moved along the DFG (non-brooding season, 421 μm sec^-1^). Note, however, that recorded velocities for various species could be influenced by temperature, algal concentration, and the kind of food used during the measurements.

In *O*. *chilensis*, both dorsal and ventral grooves are able to move food and embryos to the labial palps (S1 Video). Embryos move more quickly in the DFG than in the VFG (Fig 4). The DFG is an inner groove which is farther from the inhalant water flow, making it less prone to offspring exit and facilitating offspring mobility. Despite its high flow velocity, the transport of embryos in the DFG (S2 Video) is more frequent than for VFG. The high speeds at which embryos are moved in the DFG may be due not only to ciliary action, but to the combined action of ciliary and hydrodynamic forces, which would allow the transport of early embryos in a semi-suspensed condition above the DFG (similar to mucus slurry) (S11 Video). The rate at which the particles are transported in *Placopecten magellanicus* by mucus slurry through the DFG is also high (4.3–9 mm sec^-1^; [38]). Both *Placopecten magellanicus* and *Ostrea chilensis* have heterorhabdic type gills, which could be associated with this form of ciliary-hydrodynamic transport through the DFG, but the involvement of hydrodynamic forces in *O*. *chilensis* should be corroborated with further study. Offspring move at a more constant speed in the VFG in *O*. *chilensis*, independent of their stage of development (embryos or veligers) or the distance they move through this groove. However, embryos are unable to move long distances in contact with the surface of the VFG and many ' fall' toward the DFG.

The movement of embryos throughout the female mantle cavity is slightly different than the movement of veligers. In general, embryos move from the posterior region towards the region of the maternal palps along food grooves. However, embryos also enter a micro-cycle of movements in the interdemibranchial region. This micro-cycle includes movement of embryos on the gill lamella towards the VFG (S3 Video) (Fig 9). Some of the embryos cross the VFG and fall toward the DFG of the following interdemibanchial area. However, when most of these non-swimming embryos reach the area of the lamella near the VFG, they lose contact with the gill filaments of the lamella and ″fall″ towards the DFG of the same interdemibranchial area (S4 Video). This latter situation appears to be associated with the embryos’ insufficiently developed ciliary capabilities (e.g. absence of a velum) [27], since a similar micro-circulation pattern is not seen for later-stage veligers. The detachment of embryos from the gill filaments can be explained by the strong flow of water originating in the maternal inhalant region and directed towards the VFG. The embryos typically ″falling″ towards the DFG follow the direction of water flow, crossing through the ostia to the suprabranchial chamber. This flow facilitates contact between embryos and the gill filaments to restart the mini-cycle towards the VFG (S5 Video). This micro-circulation pattern does not involve the movement of embryos to the labial palp region, except for those that ″fall″ to the DFG and are immediately moved anteriorly.

In contrast, larvae with a well-developed velum, which includes several ciliary rings (e.g. apical, inner preoral, outer preoral and adoral cilia [27]), moved along the gill filaments of the lamella to reach the VFG, where most remained in contact with this groove and moved to the female palps (S6 Video). In this case, the water current did not cause a massive release of veligers from the VFG. In order for veligers (and also embryos) to access the VFG, they moved in close contact with the main filament towards the convex portion of the lamella, between two gill plicae (S7 Video, S8 Video). During their transport to the VFG the larvae are probably mostly in contact with the ordinary filaments from both contiguous plicae. However, if the principal filament is used there may be an energetic cost for the mother: as food particles caught in the lamella in heterorhabdic species move to the DFG using that route, the transport of those particles may be interrupted by movement of the embryos.

In general, embryos moved more rapidly in the DFG but their transport is less frequent than for veligers. Though viscous forces are important for embryos and veligers of the size—and moving at the velocity—that we measured (Reynolds number of approximately 0.1), veligers are heavier and larger than embryos, and probably move more slowly in the DFG due to their increased physical dimensions and their resulting friction with the opposing plicae. We also found that advanced-stage veligers move faster in the DFG (341.68 ±250.24 μm sec^-1^) than in the VFG (225.96 ± 86.48 μm sec^-1^), which agrees with previous research showing the same trend (DFG = 471 μm sec^-1^, VFG = 141 μm sec^-1^ in *O*. *chilensis* brooders held at 17°C; [16]).

When veligers reach the maternal palps, they swim between the palps (S9 Video) before being moved to the inhalant region by the maternal countercurrent. This veliger swimming action demonstrates the existence of a well-developed and well-ciliated velum [27]. The absence of a velum with cilia in early embryos results in the absence of such a swimming action between the female palps. Thus, after being cleaned by the female palps, embryos fall behind the female’s palps, probably reaching a dorsal area near the mouth and the mantle. This embryo cleaning process may be energetically costly for the mother for several reasons. For non-ciliated embryos that cannot swim through the palps, the mother must make additional palp movements to clean embryos. Additionally, while embryos are being cleaned, the amount of time devoted to food particle sorting at the palps will be reduced and the efficiency of that sorting will be hampered. Also, cleaning embryos at the palps may also interrupt the production of pseudofeces for particles that wouldn’t normally be ingested. Finally, the creation female counter-current may also be costly to generate and maintain, as the counter-current moves in opposition to the normal flow of particles in the mantle cavity.

The aggregations of offspring around the female palps are dispersed by a countercurrent generated by the mother (S10 Video, S11 Video). The transport velocity of veligers created by the maternal counter current exceeds the velocity of any particle (food or embryo) transported along the food grooves (Fig 11). Thus, the countercurrent is actively generated by the mother, and is also generated more often with veligers than with early embryos. Females use the gill and food grooves differently for transporting food particles and embryos, and use depends on developmental stage of the embryos. The concentration of progeny in the palp region creates a high oxygen demand that is exacerbated by the distance from the highly-oxygenated inhalant current area (Chaparro, unpubl.). Embryos have a lower oxygen demand than veligers, which could explain the lower frequency with which they are dispersed by the female countercurrent to the inhalant region, an area of higher oxygenation (Chaparro, pers. obs.).

When females of *O*. *chilensis* isolate themselves from the external environment during low-salinity events (which may last 18 hours), circulation of embryos within the mantle cavity stops after the first few minutes of isolation. Anoxic conditions can then be reached in less than 10 minutes when females are brooding advanced-stage embryos (Chaparro, pers. obs.). To avoid anoxic conditions when embryos accumulate near the female′s palps, deliberately dispersing the embryos to areas of higher oxygen such as the inhalant area should prove important for embryos. This counter current also disperses progeny to the area of the female pallial cavity where nutritious food particles enter via the pumping current. Since veligers can feed on those particles—but early-stage embryos cannot—it is more important that veligers get redispersed to that area via the maternal countercurrent so that they may feed. In some cases, however, embryos or veligers redispersed by the maternal countercurrent can be seen moving posteriorly, suspended in the inter- demibranchial space, while other embryos or veligers were simultaneously moving to the anterior region closer to the DFG (S11 Video).

Though we did not investigate the movements of embryos and food aggregates when oysters were exposed to air, females isolate themselves from the external environment, just as they do when they encounter stressful situations while submerged (e.g., low salinity). In parallel studies we found all pallial cavity activities stopped after only a few minutes of isolation. This implies that female gill cilia do not move particles or embryos during this time, so that most of the brooded progeny end up in female palp area. Ciliary movement on the velar lobes of veligers also stopped during these times; the ceasing of all of these activities is likely a response to the dramatic reduction in oxygen during this time.

It is clear from the results of this study that the brooding process within the female mantle cavity of *O*. *chilensis* confers not only a protective function, but also serves to clean mucous from the embryos, which is especially evident when females are brooding early embryo stages (S12 and S13 Video). Brooding also functions to disperse progeny through maternal countercurrents in order to avoid the generation of hypoxic areas around the palps and to make food particles available to veligers [16]. The use of parental food grooves for transporting progeny appears to explain the negative effects on the brooding females’ energy balance, since transport impairs nutrient availability to the mother during the long brooding period of this species.

Strip spawning and external larval rearing is a common hatchery practice for most oyster species. In *O*. *chilensis*, it is possible to rear feeding (post-trocophore stage) larvae stripped from the maternal brooding environment, but currently no hatchery techniques have been demonstrated for achieving successful external fertilization and rearing of zygotes outside of the mantle cavity in this species. External larval rearing has significant benefits for hatchery efficiency in aquaculture production and can greatly facilitate selective breeding. Investigations such as those presented in this paper may eventually help us to develop better methods for rearing the embryos and larvae of this species outside of the brood chamber.

## Supporting Information

S1 Video Ostrea chilensis. Veligers and food moving along VFG. VFG: ventral food groove.
(MOV)

S2 Video Ostrea chilensis. Embryos in female pallial cavity**.**
(MP4)

S3 Video Ostrea chilensis. Embryos moving along gill lamella to VFG. VFG: ventral food groove.
(MP4)

S4 Video Ostrea chilensis. Embryos micro-circulation cycle inside female pallial cavity.
(MOV)

S5 Video Ostrea chilensis. Red particles following the same micro-circulation pattern as embryos.
(MOV)

S6 Video Ostrea chilensis. Veligers moving along VFG towards female palps. VFG: ventral food groove.
(MOV)

S7 Video Ostrea chilensis. Embryos moving along principal filaments, between two plicae to the VFG. VFG: ventral food groove.
(MP4)

S8 Video Ostrea chilensis. Veligers moving along principal filaments, between two plicae to the VFG. VFG: ventral food groove.
(MP4)

S9 Video Ostrea chilensis. Veligers in between palps.
(MOV)

S10 Video Ostrea chilensis. Female counter-current dispersing of veliger to the inhalant area.
(MOV)

S11 Video Ostrea chilensis. Embryos moving in both directions simultaneously: to palps and posteriorly (female counter-current).
(MOV)

S12 Video Ostrea chilensis. Embryo cleaning process by female palps.
(MP4)

S13 Video Ostrea chilensis. Veliger cleaning process by female palps.
(MOV)
